# Multilevel analysis of early resumption of sexual intercourse among postpartum women in sub-Saharan Africa: evidence from Demographic and Health Survey Data

**DOI:** 10.1186/s12889-023-15687-8

**Published:** 2023-04-21

**Authors:** Desale Bihonegn Asmamaw, Tadele Biresaw Belachew, Wubshet Debebe Negash

**Affiliations:** 1 Department of Reproductive Health, Institute of Public Health, College of Medicine and Health Sciences, University of Gondar, P.O.Box: 196, Gondar, Ethiopia; 2 Department of Health Systems and Policy, Institute of Public Health, College of Medicine and Health Sciences, University of Gondar, Gondar, Ethiopia

**Keywords:** Early resumption of sexual intercourse, Postpartum women, Sub-Saharan Africa

## Abstract

**Background:**

Resuming sexual activity early after childbirth can cause reproductive health problems such as unwanted pregnancy, unsafe abortion, and short birth intervals, especially if contraception is not used. However, it is uncommon for healthcare providers to discuss postpartum sexual practices during prenatal and postnatal care. Therefore, this study aimed to assess early resumption of sexual intercourse and associated factors among postpartum women in sub-Saharan Africa.

**Methods:**

Secondary data analysis from the most recent Demographic and Health Surveys data from the period of 2014 to 2019/2020 of 23 countries in sub-Saharan Africa were used. A total weighted sample of 118,371 women who gave birth in the three years before the surveys were used. We analyzed the data using Stata version 14. A multilevel mixed-effect logistic regression model was fitted to identify factors associated with early resumption of sexual intercourse. Variables with a *p*-value < 0.05 in the multilevel mixed-effect logistic regression model were declared significant factors associated with the outcome variables.

**Results:**

The magnitude of early resumption of sexual intercourse among postpartum women was 67.97% (95% CI: 67.60, 68.34). Urban resident (AOR = 1.91; 95% CI: 1.83, 2.06), women with primary education 1.11 (AOR = 1.11; 95% CI: 1.07 to 1.31) and secondary education and above level 1.17 (AOR = 1.17; 95% CI: 1.09 to 1.29), husbands with primary education 1.32 (AOR = 1.32; 95% CI: 1.27, 1.38) and secondary education and above level 1.15 (AOR = 1.15; 95% CI: 1.11 to 1.25), family planning use (AOR = 95%; CI: 1.77, 1.91), fertility intention wanted then 1.24 (AOR = 1.24; 95%; CI: 1.19, 1.32) and wanted later 1.27 (AOR = 1.27; 95%; CI: 1.22, 1.46), religion (AOR = 2.08; 95%CI: 1.97, 2.17), and place of delivery (AOR = 1.51; 95%CI = 1.36, 1.65) were significantly associated with early resumption of sexual intercourse.

**Conclusion:**

The study revealed that more than two-thirds of the women had resumed sexual intercourse early after childbirth. Hence, the concerned bodies should strengthen the integration of postpartum education on sexual resumption with maternal, neonatal, and child health care services to reduce the early resumption of sexual intercourse. In addition, healthcare providers providing counseling on the resumption of postpartum sexual intercourse should focus on these factors to ensure a more effective outcome.

## Introduction

The postpartum period begins immediately after the delivery of the child and is an ideal time for interventions aimed at improving the health and survival of both mother and infant [1, 2]. During the postpartum period, women face emotional /psychological changes such as anticipation, excitement, happiness, and fulfillment, as well as anxiety, frustration, confusion, or sadness [3]. And physical changes like involution occur, lochia starts to appear, after pain, and so as well as changes in sexual function [4, 5]. Despite the many changes that childbirth brings to the sexual health and well-being of mothers, the period following childbirth has great expectations for the parents to have healthy babies [4].

Early resumption of sexual activities during the postpartum period is usually associated with reproductive tract infections and unintended pregnancies [6]. Scholars revealed that women who had initiated sexual practices early after childbirth were highly affected by puerperal infection, complications of unwanted pregnancy, unsafe abortion, and genital trauma [6, 7]. The other consequences of early resumption of sexual intercourse is short birth interval [6, 8]. Short birth intervals are associated with poor maternal health outcomes such as maternal haemorrhage, uterine rupture, low birth weight, stillbirth, early neonatal loss, and child undernutrition [6, 8]. Reduction of maternal, neonatal, and child morbidity and mortality is the primary aim of sustainable development goals. To achieve this goal, attention needs to be given to prevent unintended pregnancy, unsafe abortion, and short birth intervals as well as postpartum infection by reducing early resumption of sexual intercourse after childbirth [9, 10].

In spite of the fact that more than 90% of women worldwide want to delay or avoid future pregnancies, they usually resume sexual activity without using family planning [11]. A study conducted in Ethiopia which found that 60.41% of women resumed sexual intercourse after childbirth by 6 weeks [9], similarly, a study done in Uganda showed that 21.6% of women resumed sexual intercourse before 6 weeks after childbirth [7]. Multiple factors like socio cultural norms and beliefs, education, place of delivery, status of breast feeding, mode of delivery, and residence were some of the identified factors that can affect early resumption of sexual intercourse [4, 7, 9, 10, 12].

There has been little attention given to the issue of the resumption of sexual intercourse by researchers, policymakers, and healthcare providers even though the WHO recommends that all women be assessed 2–6 weeks after childbirth as part of a general assessment. In addition, in sub-Saharan African countries, most studies done on women's health during the postpartum period focused primarily on contraceptive utilization. There is a lack of research in this area and a lack of information on factors that affect women's early return to sexual activity after childbirth. In order to maintain the health of a family and establish smooth relationships, understanding the sexual experiences of women during the postpartum period will be essential. Hence, the current study aimed to assess the early resumption of sexual intercourse and associated factors among postpartum women in sub-Saharan Africa countries.

## Methods

The secondary data analysis was conducted based on the most recent sub-Saharan Africa (SSA) Demographic Health Surveys (DHS) datasets from 2014 to 2019/2020. These datasets were appended together to investigate the early resumption of sexual intercourse and associated factors among postpartum women in SSA.

The data were obtained from the DHS program's official database, which can be found at http://www.dhsprogram.com. Demographic and health surveys are nationally representative surveys that provide data that is comparable across countries for monitoring and impact evaluation indicators in the areas of population, health, and nutrition. The DHS uses a stratified two-stage cluster design: enumeration areas (EA) (first stage); and in each EA selected, a sample of households is drawn (second stage) [13]. We used DHS surveys done in 23 sub-Saharan African countries. For the current study, the Children’s Record (KR) data set was used, and from this data set the variable duration of post-partum abstinence after the birth of the child (m8) was used to determine the outcome variable. A weighted sample of 118,371 women who gave birth in the three years before the surveys and were interviewed for early resumption of sexual intercourse were included in the current study (Table 1).

**Table 1:** Sample size for early resumption of sexual intercourse in sub-Saharan Africa countries for each country

Countries	Year of survey	Weighted sample (n)	Weighted sample (%)
Burundi	2016/17	6458	5.46
Ethiopia	2016	5815	4.91
Kenya	2014	4638	3.92
Malawi	2015/16	7964	6.73
Rwanda	2019/20	3437	2.90
Tanzania	2015/16	4448	3.76
Uganda	2016	6453	5.45
Zambia	2018	4279	3.62
Zimbabwe	2015	2955	2.50
Angola	2015/16	5603	4.73
Cameroon	2018	4232	3.57
Chad	2014/15	9869	8.34
Benin	2017/18	7112	6.01
Gambia	2019/20	3630	3.07
Ghana	2014	2406	2.03
Guinea	2018	4050	3.42
Liberia	2019/20	1768	1.49
Mali	2018	5560	4.70
Nigeria	2018	17,593	14.86
Senegal	2019	2849	2.41
Sierra Leone	2019	4829	4.08
Lesotho	2014	1642	1.39
South Africa	2016	781	0.66
Total sample size		118,371	100

### Variables of the study

#### Dependent variable

The outcome variable for this study was the early resumption of sexual intercourse after childbirth. Early resumption of sexual intercourse after childbirth was defined as women who resumed sexual intercourse within six weeks of the postpartum period. It was categorized as 1 (early) if women resumed sexual intercourse within six weeks, and 0 (recommended time) if they resumed sexual intercourse after 6 weeks of the postpartum period [7, 14, 15].

#### Independent variables

We incorporated several individual and community-level independent variables based on reviewing different related literatures**.** Age of the women (15–24, 25–34, and 35–49), sex of child (male, female), women's education (no formal education, primary education, and secondary education and above), husband education (no formal education, primary education, and secondary and above education), religion (Christianity, Islamic, and other), place of delivery (home, health institutions), family planning use (yes, no), fertility desire (wanted no more, wanted later, and wanted then), parity (1–2, 3–4, and ≥ 5), and Amenorrhea (yes, no) were considered as individual-level variables [6, 7, 9, 16–20]**.** The household wealth index was calculated based on consumer goods such as televisions, bicycles, and cars. The material used for the roof, floor, and toilet facilities was considered in calculating the household wealth index. The wealth index was constructed using household asset data via Principal Component Analysis (PCV) to categorize individuals into wealth quintiles (poorest, poor, medium, richer and richest) [21, 22]. Regarding media exposure (yes, no), we coded yes if the women read newspaper, listened radio, or watched television for at least less than once a week, and no for otherwise [23].

Of the community level factors, residence (rural, urban) and country (East Africa; Burundi, Ethiopia, Kenya, Malawi, Rwanda, Tanzania, Uganda, Zambia, Zimbabwe: West Africa; Benin, Ghana, Guinea, Liberia, Mali, Nigeria, Sera lion, Senegal: Central Africa; Angola, Cameroon, Chad and South Africa; Lesotho and South Africa) were directly accessed from the EDHS data set. However, the aggregate community level independent variables (community level poverty, community level media exposure, and community level education) were constructed by aggregating individual-level characteristics at the community (cluster) level. They were categorized as high or low based on the distribution of the proportion values computed for each community after checking the distribution by using the histogram. The aggregate variable was not normally distributed, and the median value was used as a cut-off point for the categorization [9, 22].

### Data processing and statistical analysis

The data were extracted from recent DHS data sets and cleaned, recorded, and analyzed with STATA 14 Statistical Software. We denormalized the women's individual weight variable by dividing the women’s individual standard weight by the sampling fraction of the included survey (calculated by dividing the number of samples included by the total eligible population of that country), as we have used DHS data from many countries. We used the weighting variable (v005) as a relative weight normalized to make the analysis survey-specific, while for the pooled data, we denormalized the women’s individual standard weight variable by dividing the women’s individual standard weight by the sampling fraction of each country. The formula for the denormalized weight variable is: female adjusted weight = V005 × (total females aged 15–49 years in the country at the time of the survey) / (number of women aged 15–49 years interviewed in the survey). Regarding the clustering effect, we applied a three-level multilevel model for identifying the factors using the enumeration area (EA) and the country as a random variable, and then we checked whether there was significant clustering at each level. The ICC and LR test at the third level (country level) was not significant, unlike the EA, which is why we have applied the two-level multilevel modeling. Descriptive statistics were described using frequencies, percentages, median, and interquartile range and were presented using tables, figures, and narratives.

### Random effects (measure of variation)

Intra-class correlation coefficient (ICC) and Proportional Change in Variance (PCV) were computed to measure the variation between clusters. Taking clusters as a random variable, the ICC reveals the variation of early resumption of sexual intercourse between clusters is calculated as; $ICC=\frac{VA}{VA+3.29}*100\%$. Moreover, the PCV reveals the variation in the magnitude of early resumption of sexual intercourse among postpartum women explained by factors and calculated as; $PCV=\frac{Vnull-VA}{V null}*100\%$ where; Vnull = variance of the initial model, and VA = area/cluster level variance [24].

### The fixed effects (measure of association)

The fixed effects or measure of association was used to estimate the association between the likelihood of magnitude of early resumption of sexual intercourse, individual, and community levels independent variables. It was assessed and the strength was presented using Adjusted Odds Ratio (AOR) and 95% confidence intervals with a *p*-value of < 0.05.

$$Log \left(\frac{\pi ij}{1-\pi ij}\right)=\beta o+ \beta 1xij+ \beta 2xij+\dots uj+eij$$

where, $\pi ij$: the probability of early resumption of sexual intercourse, $1-\pi ij$: the probability of sexual resumption on the recommended time. ß0 is intercept that is the effect of early resumption of sexual intercourse when the effect of all independent variables is absent. $\beta 1xij$ are individual and community level variables for the i^th^ individual in group j, respectively. The ß’s are fixed coefficients indicating a unit increase in X can cause a ß unit increase in probability early resumption of sexual intercourse. The uj shows the random effect for the j^th^ clusters [24, 25].

### Model building for multi-level analysis

Model comparisons were done using the deviance test and log likelihood test and the model with the highest log-likelihood ratio and the lowest deviance was selected as the best-fitted model. Moreover, multicollinearity was tested using the variance inflation factor (VIF) and we have got a VIF of less than five for each independent variable with a mean VIF of 1.67, indicating there was no significant multicollinearity between independent variables. First bi-variable multilevel logistic regression analysis was performed, and those variables with a *p*-value of < 0.25 were considered in the multivariable analysis. After selecting variables for multivariable multilevel analysis, four models were fitted; the null model (without independent variables), mode I (containing only individual-level factors), mode II (community-level factors), and model III (containing both individual and community-level factors). Variables with a *p*-value < 0.05 in the multilevel mixed-effect logistic regression model were declared as significant factors associated with early sexual resumption after childbirth.

## Results

### Socio-demographic related factors

A total weighted sample of 118,371 postpartum women were included in this analysis. The median age of the study participants was 28 years (IQR: 23–33). About 48.25% of participants fell within the age category of 25–34 years, and 70.06% of the women were rural dwellers. Two-fifths (40.49%) of the participants had no formal education. About 70.30% of participants were Christian religious followers (Table 2).

**Table 2:** Socio demographic related factors of the participants in sub-Saharan Africa countries

Variables	Weighted frequency	Percentage (%)
Women age		
15–24	37,458	31.64
25–34	57,119	48.25
35–49	23,794	20.10
Women education		
No formal education	47,928	40.49
Primary education	39,284	33.19
Secondary education and above	31,159	26.32
Women occupation		
Working	80,654	68.14
Not working	37,717	31.86
Husband education		
No formal education	40,135	33.91
Primary education	34,836	29.43
Secondary education and above	43,400	36.66
Wealth index		
Poor	53,914	45.55
Middle	23,570	19.91
Rich	40,887	34.54
Media exposure		
Yes	72,510	61.26
No	45,861	38.74
Religion		
Christianity	83,214	70.30
Islamic	31,245	26.40
Other	3912	3.31
Resident		
Rural	82,929	70.06
Urban	35,442	29.94
Community media exposure		
Lower	55,142	46.58
Higher	63,229	53.42
Community-women education		
Lower	59,166	49.98
Higher	59,205	50.02
Community level poverty		
Lower	56,814	48.00
Higher	61,557	52.00
Sex of the child		
Male	60,355	50.99
Female	58,016	49.01

### Obstetric related factors

About 70% and 64.59% of the women used family planning and gave birth at health institutions, respectively. More than three-fourths (79.52) of the respondents had exclusive breastfeeding (Table 3).

**Table 3:** Obstetric related factors of the respondent in sub-Saharan Africa countries

Variables	Frequency	Percentage
Parity		
1–2	41,235	34.84
3–4	46,791	39.53
≥ 5	30,345	25.64
Amenorrheic		
Yes	49,235	41.59
No	69,136	58.41
Family planning use		
Yes	35,510	70.00
No	82,861	30.00
Place of delivery		
Home	41,915	35.41
Health institutions	76,456	64.59
Exclusive breast feeding		
Yes	94,133	79.52
No	24,238	20.48
Fertility desire		
Wanted then	18,908	15.97
Wanted later	56,154	47.44
Wanted no more	43,309	36.59

### Magnitude of early resumption of sexual intercourse

**Fig. 1:**
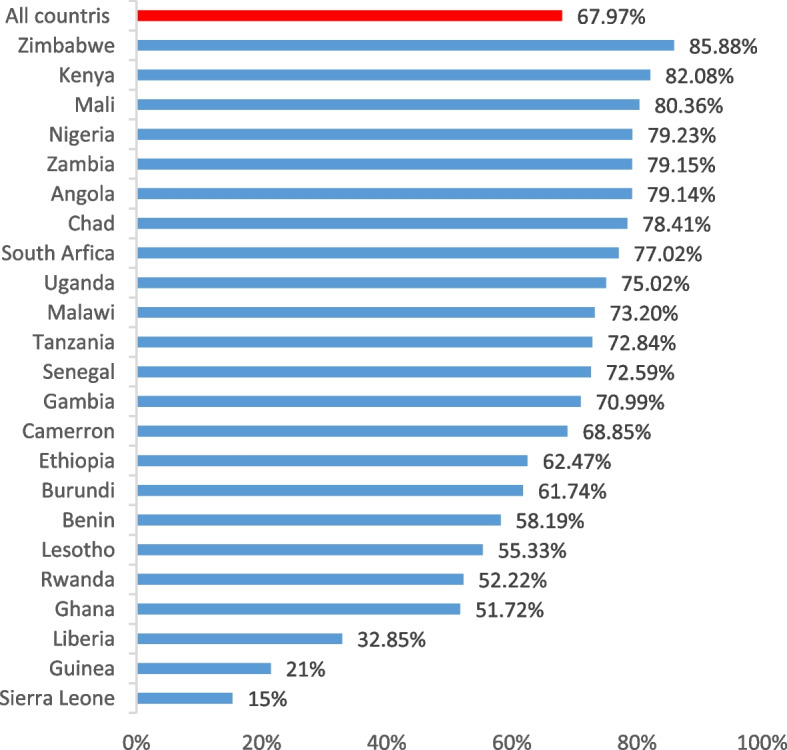
Early resumptions of post-partum sexual intercourse in sub-Saharan Africa countries

Overall, 67.97% (95% CI: 67.60, 68.34) of the postpartum women had an early resumption of sexual intercourse. Of the 23 countries, Zimbabwe and Sierra Leone accounted for the highest (85.88%) and the lowest (15.00%) early resumption of sexual intercourse, respectively (Fig. 1).

### Factors associated with early resumption sexual intercourse

Early resumption of sexual intercourse varied significantly across clusters. In the baseline model without an independent variable, 54.36% of the variance in the early resumption of sexual intercourse was explained by the variation in characteristics between clusters (ICC = 0.5436). In model 3, which included both individual and community-level factors, the between-cluster variation was reduced to 38.71%. Accordingly, the variance in the early resumption of sexual intercourse could be explained by differences in clusters. Moreover, deviance was used to assess model fitness; hence, the model with the lowest deviance (model III) value was found to be the best-fitted model (Table 4)**.**

**Table 4:** Model comparison and random effect analysis result in sub-Saharan Africa countries

Parameters	Null model	Model I	Model II	Model III
Variance	0.89	0.67	0.29	0.13
ICC (%)	54.36	54.21	41.71	38.41
PCV (%)	Ref	24.72	67.42	85.39
Log-likelihood	-73,294.30	-73,283.88	-69,199.81	-69,192.13
Deviance	146,588.60	146,567.76	138,399.62	138,384.26

In the multilevel multivariable logistic regression model, women education, husband education, residence, place of delivery, family planning use, religion, and fertility desire were statistically associated with early resumption of sexual intercourse.

The odds of resuming sexual intercourse early were 1.11 (AOR = 1.11; 95% CI: 1.07 to 1.31) larger among women who attended primary education and 1.17 (AOR = 1.17; 95% CI: 1.09 to 1.29) times larger among women who attended secondary education than women with no formal education. The odds of resuming sexual intercourse early were 1.32 (AOR = 1.32; 95% CI: 1.27, 1.38) times larger among women who attended primary education and 1.15 (AOR = 1.15; 95% CI: 1.11 to 1.25) times larger among women who attended secondary education than women with no formal education. The odds of resuming sexual intercourse early were 1.91 (AOR = 1.91; 95% CI: 1.83, 2.06) times larger among women who live in urban areas compared to women who live in rural areas. The odds of resuming sexual intercourse early were 2.08 (AOR = 2.08; 95%CI: 1.97, 2.17) times larger among women who followed the Islamic religion compared to those who followed the Christian religion. The odds of resuming sexual intercourse early were 1.51 (AOR = 1.51; 95%CI = 1.36, 1.65) times larger among women who were delivered at institution compared to those who were delivered at home.

Likewise, the odds of resuming sexual intercourse early were 1.84 (AOR = 95%; CI: 1.77, 1.91) times larger among respondents who have used family planning as compared to those who have not used family planning. The odds of resuming sexual intercourse early were 1.24 (AOR = 1.24; 95%; CI: 1.19, 1.32) times larger among women who wanted then and 1.27 (AOR = 1.27; 95%; CI: 1.22, 1.46) times larger among women who wanted later as compared to those who had not wanted any more children. The odds of resuming sexual intercourse early were 2.02 (AOR = 2.02; 95%; CI: 1.92, 2.23) times larger among women from Central African countries and 1.49 (AOR = 1.49; 95%; CI: 1.44, 1.54) times larger among women from East African countries as compared with women from West Africa countries (Table 5).

**Table 5:** Multivariable multilevel logistic regression model results of early resumption of sexual intercourse in sub-Saharan Africa

Variables	Model I (AOR, 95%CI)	Model II (AOR, 95%CI)	Model III (AOR, 95%CI)
Age of the respondent			
15–24	1.13 (1.06, 1.24)		1.13 (0.97, 1.32)
25–34	1.11(1.01, 1.25)		1.05 (0.94, 1.23)
35–49	1		1
Sex of child			
Female	1		1
Male	1.61 (1.47, 1.79)		1.56 (0.97, 2.03)
Women education			
No formal education	1		1
Primary education	1.32 (1.27, 1.38)		1.11 (1.07, 1.31)^*^
Secondary education and above	1.13 (1.09, 1.18)		1.17 (1.09, 1.29)^*^
Husband education			
No formal education	1		1
Primary education	1.17 (1.06, 1.28)		1.32 (1.27, 1.38)^*^
Secondary education and above	1.13 (1.08, 1.19)		1.15 (1.11, 1.25)^*^
Wealth index			
Poor	1		1
Middle	1.11 (0.97, 1.21)		1.04 (0.99, 1.11)
Riche	1.32 (1.25, 2.39)		1.26 (0.93, 1.41)
Religion			
Christianity	1		1
Muslim	2.07 (1.95, 2.17)		2.08 (1.97, 2.17)^*^
Other	1.14 (1.08, 1.19)		1.03 (0.94, 1.13)
Place of delivery			
Home	1.52 (1.46, 1.57)		1.51 (1.36, 1.65)^*^
Health institution	1		1
Family planning use			
No	1		1
Yes	1.84 (1.77, 1.91)		1.84 (1.77, 1.91)^*^
Fertility desire			
Wanted no more	1		1
Wanted later	1.24 (1.19, 1.28)		1.27 (1.22, 1.46)^*^
Wanted then	1.28 (1.21, 1.33)		1.24 (1.19, 1.32)^*^
Parity			
1–2	1.25 (1.08, 1.41)		1.26 (0.98, 1.45)
3–4	1.19 (1.03, 1.31)		1.21 (0.93, 1.71)
≥ 5	1		1
Amenorrhea			
Yes	1		1
No	1.41 (1.24, 1.67)		1.52 (0.94, 1.79)
Resident			
Rural		1	1
Urban		2.13 (1.98, 2.25)	1.91 (1.83, 2.06)^*^
Community media exposure			
Lower		1	1
Higher		0.94 (0.89, 1.08)	1.06 (0.95, 1.09)
Community-women education			
Lower		1	1
Higher		1.05 (0.98, 1.11)	1.07 (0.98, 1.61)
Community-level poverty			
Lower		1	1
Higher		0.98 (0.91, 1.07)	1.04 (0.89,1.12)
Country			
West Africa		1	1
East Africa		1.65 (1.59, 1.72)	1.49 (1.44, 1.54)
South Africa		1.11 (0.99, 1.22)	0.92 (0.84, 1.06)
Central Africa		2.18 (2.09, 2.27)	2.02 (1.92, 2.23)

*AOR* Adjusted odds ratio, Model 1: adjusted for individual-level factors, Model 2: adjusted for community-level factors, Model 3: adjusted for both individual and community-level factors (full model), ^*^statistically significant at *p*-value < 0.05 in the full model

## Discussion

The aim of this study was to determine levels of early resumption of sexual intercourse and identify associated factors among postpartum women in SSA. This study found that 67.97% (95% CI: 67.60, 68.34) of the postpartum women had early resumption of sexual intercourse. This finding is higher than the previous studies conducted in Uganda [7], China [26], Nigeria [27], and United States 43% [28]. And also it is lower than studies done in Ethiopia [4, 6, 9, 14]. These variations might be explained by differences in religious practices, norms, sociocultural beliefs, and sexual attitudes among women worldwide [29]. Early resumption of sexual intercourse is influenced by subjective norms, social beliefs, and values held by society about the early resumption of sexual intercourse after childbirth [29, 30]. Moreover, this might be due to the difference in background characteristics. For instance, the proportion of women who used family planning and women who had no formal education in this study were 70% and 40.5% and in Uganda, it was 26.5% and 2.9% [7], respectively. In addition, a study conducted in Nigeria revealed that only 7.2% [27] of women had no formal education, which was lower than that of the current study (40.5%). Previous studies documented that education and family planning had a negative and positive relationship with early resumption of sexual intercourse, respectively [4, 7, 9].

The current study showed that women who live in urban areas were more likely to have an early resumption of sexual intercourse than their counterparts. This finding is in line with studies done in Northwest Ethiopia [4], Nigeria [31], and Eswatini [29]. This could be because women living in urban areas are less likely adhere to and respect traditional practices, religious beliefs, and the cultural practice of sexual abstinence during the postpartum period [29, 32]. Moreover, urban women having more access to more health information thereby influencing their decision [14].

Women with formal education were more likely to resume sexual intercourse early as compared to women with no formal education. This finding was coherent with the studies conducted in Ethiopia [9] and Uganda [7]. The reason could be that educated women do not marry at a younger age and do not have as many children as they would like. Hence, they might need to have children and they need to start sexual intercourse early [33]. Moreover, This might be due to that educated women have a good opportunity of getting more information and counselling from health professionals on exclusive breast feeding and postpartum family planning use, so that they can initiate postpartum family planning on recommended time. Studies reported postpartum family planning use as one of the factors associated with early postpartum resumption of sexual intercourse [7, 26]. Women with husbands who attended formal education were more likely to resume sexual intercourse early compared with their counterparts. This study was supported by studies conducted in Uganda [7, 34]. This could be because husband who is educated could get more information on early resumption of sexual intercourse including misconceptions and can help women to practice sexual intercourse with no fear of physiological change [4]. In addition, educated husbands recommend that women use family planning during the postpartum period.

Women who were using family planning were at higher odds of resuming early sexual intercourse than those who did not use it. This finding is consistent with other previous studies done in Ethiopia [4] and Uganda [7]. It might be because women expected themselves to have achieved the recommended birth spacing intervals and to be free from the risk of unintended pregnancy.

Likewise, the odds of early resumption of sexual intercourse were significantly higher among participants who had delivered at the health institutions as compared to women who had delivered at home. This could be because women who have delivered at health institutions might possibly have a better opportunity to receive counseling and information from healthcare professionals about exclusive breastfeeding and postpartum family planning so that they can use postpartum family planning at the recommended time. Researchers discovered that the use of family planning is one of the factors associated with the early resumption of sexual activities [4, 7]. Furthermore, women who had given birth at home were usually less educated and most of them lived in rural areas [35, 36]. Similarly, the resumption of sexual intercourse among postpartum women who live in Central and East Africa countries was more likely higher than those of postpartum women who live in West Africa countries. The possible justification might be due to the difference in sociodemographic characteristics and cultural variations.

In this study, women who wanted to have another child were more likely to resume sexual intercourse early after childbirth. The finding is congruent with previous evidence from Ethiopia [14], Nigeria [27], and Australia [37]. This might be because when women resume sexual intercourse after childbirth, they may know that they are at risk for pregnancy, which may fulfil their desire for another child. Similarly, religion influences the timing of the resumption of sexual intercourse. Postpartum women who follow the Islamic religion were more likely to resume sexual intercourse early after childbirth compared to those who follow the Christian religion. This might be According to the Christian religion, if a woman gives birth to a female, the woman must abstain for up to 80 days, and if a woman gives birth to a male, the woman must abstain for up to 40 days.

## Strengths and limitations of the study

This study used nationally representative data sets, which were collected with standardized and validated data collection tools. A multilevel analysis was used in this study to account for the hierarchical nature of the data. The cross-sectional nature of the survey does not show the causal relationship between outcome and independent variables. The study relies on participants' self-reports and their willingness to provide accurate information, so it might not provide an objective measure of the time of resumption of sexual activities. Furthermore, the topic is sensitive, and some respondents may have been reluctant to reveal their real sex life.

## Conclusion

The study revealed that more than two-thirds of the women had resumed sexual intercourse early after childbirth. This implies that a substantial proportion of women could be at risk of puerperal infection, short birth interval, high rates of unintended pregnancy, unsafe abortion and an increased risk of genital trauma. Particularly when they delay the initiation of postpartum family planning and the intensity of exclusive breastfeeding cannot be guaranteed. This raises the risk of maternal and child morbidity and mortality. Women's education, husband's education, residence, religion, place of delivery, fertility desire, family planning use, and countries were significantly associated with the early resumption of sexual intercourse. Hence, the concerned bodies should strengthen the integration of postpartum sexual education with maternal, neonatal, and child health care services to reduce the early resumption of sexual intercourse. In addition, healthcare providers providing information on the resumption of postpartum sexual intercourse should focus on these factors to ensure a more effective outcome.

## Data Availability

In this study, we used the most recent Demographic and Health Survey data, which is freely available online at (https://www.dhsprogram.com).
